# Insights on the concept of indicator populations derived from parentage‐based tagging in a large‐scale coho salmon application in British Columbia, Canada

**DOI:** 10.1002/ece3.6383

**Published:** 2020-05-18

**Authors:** Terry D. Beacham, Colin Wallace, Kim Jonsen, Brenda McIntosh, John R. Candy, David Willis, Cheryl Lynch, Ruth E. Withler

**Affiliations:** ^1^ Fisheries and Oceans Canada Pacific Biological Station Nanaimo BC Canada; ^2^ Fisheries and Oceans Canada Regional Headquarters Vancouver BC Canada

**Keywords:** coded‐wire tags, coho salmon, fishery management, genetic stock identification, genotyping by sequencing, indicator populations, parentage‐based tagging

## Abstract

For Pacific salmon, the key fisheries management goal in British Columbia (BC) is to maintain and restore healthy and diverse Pacific salmon populations, making conservation of salmon biodiversity the highest priority for resource management decision‐making. Salmon status assessments are often conducted on coded‐wire‐tagged subsets of indicator populations based on assumptions of little differentiation within or among proximal populations. In the current study of southern BC coho salmon (*Oncorhynchus kisutch*) populations, parentage‐based tagging (PBT) analysis provided novel information on migration and life‐history patterns to test the assumptions of biological homogeneity over limited (generally < 100 km) geographic distances and, potentially, to inform management of fisheries and hatchery broodstocks. Heterogeneity for location and timing of fishery captures, family productivity, and exploitation rate was observed over small geographic scales, within regions that are, or might be expected to be, within the area encompassed by a single‐tagged indicator population. These results provide little support for the suggestion that information gained from tagged indicator populations is representative of marine distribution, productivity, and exploitation patterns of proximal populations.

## Graphical abstract

Salmon status assessments are often conducted on coded‐wire‐tagged subsets of indicator populations based on the assumptions of little differentiation within or among proximal populations. In the current study of southern BC coho salmon (*Oncorhynchus kisutch*) populations, parentage‐based tagging (PBT) analysis provided novel information on migration and life‐history patterns to test the assumptions of biological homogeneity over small geographic distances and, potentially, to inform management of fisheries and hatchery broodstocks.

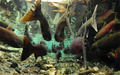

## 1 INTRODUCTION

Pacific salmon populations in British Columbia (BC) have been organized into conservation units (CUs) for management believed to constitute the basic units of intraspecific biodiversity that should be conserved for sustainability over time (Holtby & Ciruna, 2007). Salmon management at the individual CU, or aggregated CU (management unit; MU), level is expected to provide long‐term persistence, resilience, and harvest opportunity in the face of multiple challenges including habitat destruction, climate change, aquaculture, and hatchery supplementation (Fisheries and Oceans Canada (FOC) 2005, 2018). Current management of coho salmon (*Oncorhynchus kisutch*) in BC is based on life history, marine distribution, and exploitation information obtained from coded‐wire‐tagged (CWT) “indicator” populations, from which a portion of individuals carries a physical tag that can be recovered from a fish for identification purposes. Tagged coho salmon indicator assessment programs, and the geographic boundaries for populations that each indicator was intended to represent, were initiated over 40 years ago, predating the development of genomic technologies to assist in characterization of biodiversity and the identification of coho salmon CUs in BC. The concept of an indicator population is fundamental to the current CWT program applied to assessment and management of coho and Chinook salmon (*O. tshawytscha*) in both Canada and the United States of America.

Indicator populations are directly assessed and deemed representative of other populations in the area that are not directly assessed. Hatcheries in BC have maintained indicator release groups, defined by one or more release strategies for a population, to support hatchery operations through estimation of biostandard references for marine survival, exploitation rate, and catch distribution, as well as fecundity and age‐class distribution. Indirect assessment uses biostandards, where average values for assessment outputs such as survival rate from an indicator population of the same species are used to estimate production. Biostandards calculated for a group of fish can be adjusted for application to another group of fish depending on variations in parameters such as release stage, migration patterns, and harvest effort between the two hatchery release groups. For example, Robertson Creek and Conuma River are two populations on the west coast of Vancouver Island, with the Robertson Creek population currently marked with CWTs and considered the indicator population, and the Conuma River population marked with CWTs during 1979–2002. Current estimates of harvest of the Conuma River population are derived from the current Robertson Creek harvest, with biostandards developed for marine survival and catch distribution derived when both populations were marked with CWTs. Biostandards have been refined over time to represent hatchery production areas. CWT indicator regions and CUs are not well‐aligned, and many CUs, or even MUs, lack tagged indicator populations. Delivery of CU‐based salmon management for biodiversity and sustainability would benefit from an updated consideration of the assessment and management tools now available to characterize, monitor, and conserve biodiversity (Benestan et al., 2016; Hunter, Hoban, Brugord, Segelbacher, & Bernatchez, 2018).

The concept of using indicator populations is based on an expectation of biological homogeneity over the geographic region represented by the indicator population. Often, indicator populations are hatchery‐supplemented and only a portion of the hatchery‐origin component of the population is tagged, and is assumed to be representative of the entire population, both of hatchery origin and natural origin. The indicator population is expected to be representative, in terms of life history, marine distribution, productivity, and exploitation pattern, of other populations within the indicator region. In particular, tagged indicator populations are used to estimate exploitation rates and fishery contributions. The lack of sufficient and appropriate tagged indicator populations for Canadian populations can produce questionable estimates of stock proportions for fishery harvest when the estimates are based solely on adipose‐fin‐clipped or adipose‐fin‐tagged individuals. For example, in the July 2017 sample from the Johnstone Strait creel survey obtained through direct catch sampling, coho salmon from two CUs (Southern Coastal Streams–Queen Charlotte Strait–Johnstone Strait–Southern Fjords, Homathko–Klinaklini Rivers) comprised 31% of the sample, but samples delivered to a central processing laboratory for potential CWT recovery from adipose‐fin‐clipped individuals for the same time period displayed no contribution of these two CUs to the sample (Beacham et al., 2019a).

Recent and ongoing improvements in genetic and genomic analytical methods indicate that a genetic‐based approach may provide the most informative, viable, and cost‐effective methodology for assessment and management of hatchery origin and wild Pacific salmon (Beacham et al., 2019a, 2020). Anderson and Garza (2006) noted that parentage‐based tagging (PBT) provided a method of identifying both the age and population of origin for individual salmon. PBT uses molecular‐based approaches to conduct large‐scale parentage assignments and has resulted in the unprecedented ability to identify genetically millions of hatchery‐origin salmonids to their hatchery of release and age (Steele, Hess, Narum, & Campbell, 2019). Steele et al. (2013), Hess et al. (2016), and Steele et al. (2019) demonstrated the accuracy and utility of PBT for the management of steelhead trout (*O. mykiss*) in the Snake River basin and the upper portions of the Columbia River drainage. Beacham et al. (2017, 2018) demonstrated the potential of a PBT‐genetic stock identification (GSI) approach for identification of individual coho salmon and Chinook salmon (*O. tshawytscha*) to specific hatcheries in BC, even with geographically diverse mixtures of populations present in mixed‐stock fishery samples.

Beacham et al. (2019a) provided evidence that PBT‐GSI‐based assessment and management of hatchery origin and wild coho salmon were a practical approach, as demonstrated by a large‐scale application to fisheries management and assessment in BC. Population‐ and family‐specific distributions among fisheries, origins, and productivity of hatchery broodstocks and associated stray rates among populations were also evaluated via PBT (Beacham et al., 2019b). As well as providing improved estimates of stock composition from mixed‐stock fisheries, PBT allows evaluation of family‐specific productivity and exploitation, evaluation of productivity in specific components (differentiated by life‐history type, spawn times, or rearing environment) of a hatchery broodstock, assessment of hatchery–wild interactions in salmonids (Araki, Berejikian, Ford, & Blouin, 2008, McClure et al., 2008, Jones & Wang, 2010), and the genetic basis for salmon migration and marine residency. Direct DNA sequencing is powering a revolutionary application of genetics to fisheries management and assessment, providing cost‐effective genotyping at hundreds of single nucleotide polymorphism (SNP) loci (Campbell, Harmon, & Narum, 2015) or tens of microsatellites (Bradbury et al., 2018). Harnessing the evolving power of genetic and genomic technologies will provide ongoing insight into the adaptive variation at the heart of biodiversity, and the impacts on wild populations resulting from fishery and hatchery broodstock activities as well as climate change and changing ocean regimes (Bernatchez et al., 2017).

In the current study, PBT methodology based on variability at 304 single nucleotide polymorphisms (SNPs) was applied to coho salmon sampled from fisheries and escapements (including both hatchery broodstock and nonbroodstock) in BC. Commercial and recreational coho salmon fisheries were sampled in 2017 and 2018, and hatchery broodstock and nonbroodstock river escapements (2016 and 2017 only) were sampled in 2016, 2017, and 2018 to identify progeny contributions from individual parents of 2014 and 2015 hatchery broodstocks. Complete broodstock genotyping of 20 hatchery‐enhanced populations in 2014 involved genotyping 6,061 individuals. There were subsequently 21,195 individuals genotyped from fishery, hatchery brood, and escapement sampling in 2016 and 2017 in order to analyze 2014 parental contributions to the harvest, hatchery, and river returns. In 2018, 14,124 individuals were genotyped in fishery and hatchery broodstocks in order to evaluate 2015 parental contributions to harvest and hatchery broodstocks. Information on geographic variability among populations in fishery distribution and timing of catch, and family productivity and exploitation rates was generated. Our analysis of within and among population variability did not support the premise that indicator populations display abundance, distribution, and timing characteristics that are representative of other populations within management units based on geographic proximity.

## 2 METHODS

### 2.1 Fishery sample collection

**FIGURE 1:**
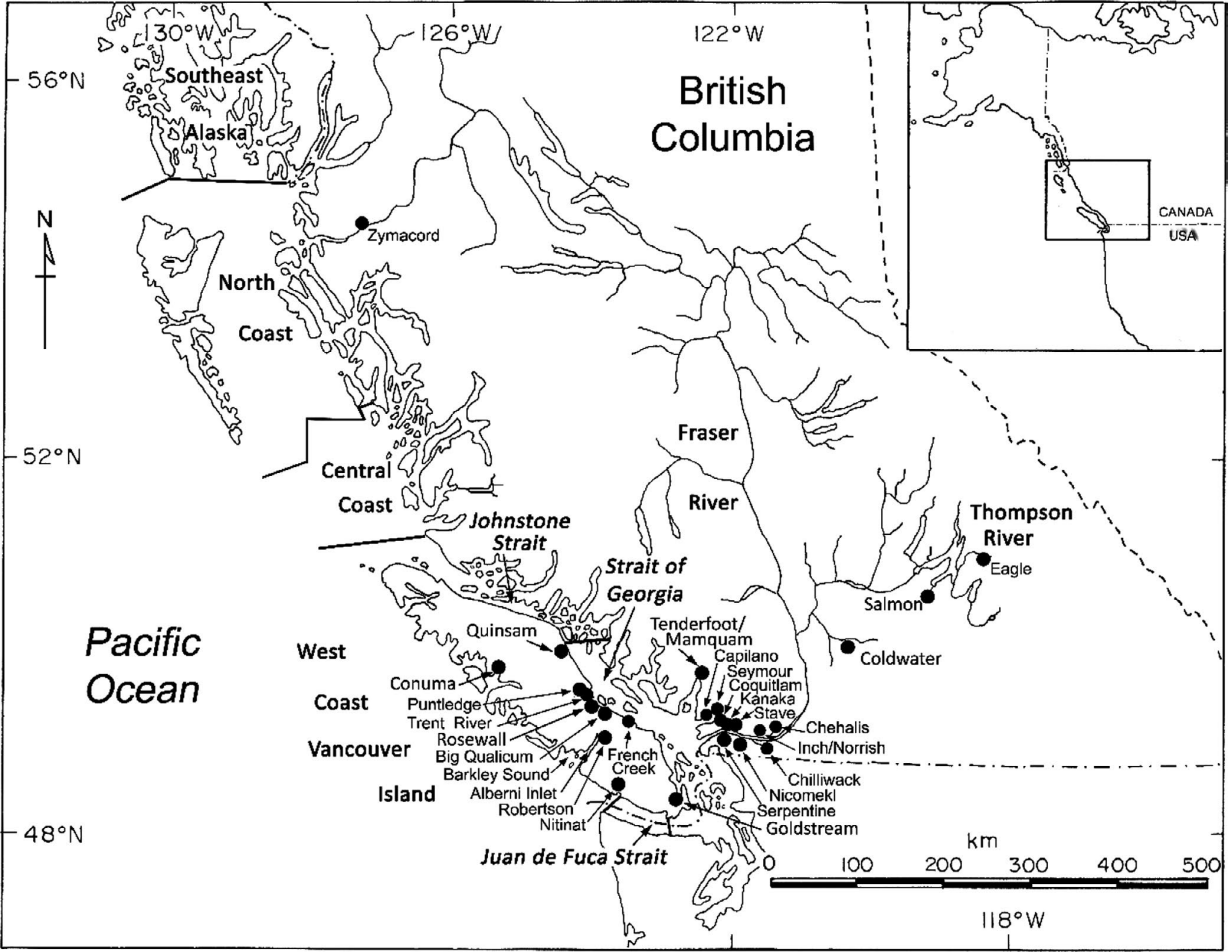
Map indicating geographic locations for fishery sampling and 26 populations for which parentage‐based tagging was applied to identify individuals in both fishery and escapement sampling

Six fishery areas were defined for coho salmon sampled from fisheries conducted in BC during 2017 and 2018. The fishery areas were as follows: North Coast (NC), Central Coast (CC), Johnstone Strait (JS), Strait of Georgia (SOG), Juan de Fuca Strait (JDF), and west coast of Vancouver Island (WCVI) (Figure 1). Samples from commercial, recreational, and First Nations fisheries within a fishery area were pooled, and samples from Barkley Sound and Alberni Inlet were pooled with WCVI samples. Further details on fishery sampling were outlined by Beacham et al. (2019a). There were 1,499 PBT identifications made in 2017 fishery samples (Beacham et al., 2019a) and 1,792 PBT identifications made in 2018 fishery samples, and these 3,291 PBT identifications comprised the fishery pool used to identify individuals to specific parents in either the 2014 for 2015 hatchery broodstocks.

### 2.2 Escapement and broodstock sample collection

Twenty hatchery broodstocks were sampled in 2014, and 6,061 individuals were genotyped in these broodstocks (Beacham et al., 2017). The hatchery broodstocks originated from the Lower Fraser River, southern BC mainland, and Vancouver Island, and constituted the parental base for subsequent PBT assignments in 2016 and 2017 fishery and escapement sampling.

In 2015, 31 hatchery broodstocks were sampled, and 7,431 individuals were genotyped in these broodstocks. The number of hatchery broodstock sampled had increased relative to 2014, and these broodstocks constituted the parental base for PBT assignments of jacks in the 2017 broodstock or escapement samples, as well as the base for adult assignments in the 2018 fisheries.

In 2016, adipose‐fin‐clipped jacks (aged 2 years) were sampled from nonbroodstock escapement as outlined by Beacham et al. (2019a), and 543 jacks were assigned to parents via PBT. Additionally, individuals in the 2016 hatchery broodstocks were genotyped (6,646 individuals) and assessments conducted to determine whether any individuals in the 2016 broodstocks could be matched to parents in the 2014 broodstocks via PBT. There were 143 PBT identifications made of jacks in the 2016 broodstocks.

In 2017, escapements (nonbroodstock hatchery and river returns, clipped individuals only) from 13 populations (1,692 individuals genotyped, 1,530 PBT identifications) were sampled with the objective of evaluating straying rate among populations (Beacham et al., 2019a). Hatchery broodstocks were sampled for 34 populations (7,251 individuals genotyped, 4,447 PBT identifications), of which PBT analysis was used to assign individuals to 19 of the broodstocks sampled, as broodstock sampling has to occur three years previously before PBT identifications can be made on adult (age 3) returns.

In 2018, 40 hatchery broodstocks were sampled (7,805 individuals genotyped, 4,406 PBT identifications), of which PBT analysis could be used to assign individuals to 31 broodstocks, as these broodstocks had been genotyped in 2015. In total, 21,702 individuals (8,996 PBT identifications) were genotyped in hatchery broodstocks during 2016–2018, and 2,265 individuals (2,073 PBT identifications) were genotyped in nonbroodstocks escapements during the same period. These 11,261 PBT identifications constituted the escapement pool used to identify individuals to specific parents in either the 2014 for 2015 hatchery broodstocks. The populations included in the analyses are outlined in Table 1.

**TABLE 1:** Coho salmon spawning locations, sample collection years, and total number of fish sampled for 27 populations in nine conservation units in British Columbia

Conservation Unit	Population	Years	Number
Lower Skeena	Zymacord River	1996, 2015–2018	61
Howe Sound–Burrard Inlet	Capilano River	2014–2018	3,725
	Mamquam River	2014–2018	264
	Seymour River	2015–2018	711
	Tenderfoot Creek	2014–2018	1,195
East Vancouver Island–Georgia Strait	Big Qualicum River	2014–2018	2,405
	French Creek	2015, 2017, 2018	117
	Goldstream River	2014–2016, 2018	715
	Puntledge River	2014–2018	3,669
	Quinsam River	2014–2018	3,039
	Rosewall Creek	2014–2018	487
	Trent River	2015, 2017, 2018	86
West Vancouver Island	Conuma River	2014–2018	809
	Robertson Creek	2014–2018	1,432
Juan de Fuca–Pachena	Nitinat River	2014–2018	2,509
Lower Fraser River	Chehalis River	2014– 2018	2,145
	Chilliwack River	2014–2018	4,765
	Coquitlam River	2015–2018	273
	Inch Creek	2006, 2009, 2012, 2014–2018	2,351
	Kanaka Creek	2005, 2015–2018	234
	Norrish Creek	2001, 2014–2018	720
	Stave River	2014–2018	870
Lower Thompson	Coldwater River	2014–2018	685
South Thompson	Eagle River	2015–2018	321
	Salmon River	2008, 2010, 2014–2018	241
Boundary Bay	Nicomekl River	2014–2018	348
	Serpentine River	2014–2018	574

Note

*N* is the number of fish genotyped in the population.

In brief summary, Beacham et al. (2019a) previously outlined PBT fishery assignments for 2017 fisheries, and PBT assignments for 2016 jack escapement samples, with all PBT assignments made to parents in the initial 2014 hatchery broodstocks that were genotyped. Beacham et al. (2019b) previously outlined jack PBT assignments for the 2016 hatchery broodstocks, and jack and adult PBT assignments for the 2017 hatchery broodstock and escapement samples, with PBT assignments made to parents in the 2014 and 2015 hatchery broodstocks. The current study incorporates this previous information and adds to it the 2018 fishery PBT assignments and the 2018 hatchery broodstock and escapement PBT assignments, with PBT assignments made to parents in the 2014, 2015, or 2016 hatchery broodstocks.

### 2.3 Genotyping

The detailed procedure for library preparation and genotyping was outlined by Beacham et al. (2017) and a summarized version provided by Beacham et al. (2019a). Summarized briefly, 768 individuals with up to 304 amplicons per individual were loaded on a P1 chip v3 with an Ion Chef, two chips were loaded consecutively with one run of the Ion Chef, both chips were then subsequently loaded on to an Ion Torrent Proton sequencer, and the genotype of each individual recorded with automated scoring of the genotype via Proton software Variant Caller® at one SNP site in each amplicon. Genotypes at all available SNPs for an individual were assembled to provide a multilocus individual genotype. This multilocus genotype was the basic input for PBT analysis.

### 2.4 Identification of individuals

PBT was used to identify individuals in fishery and escapement samples by matching the genotype of the individual to the genotypes of prospective parents (COLONY, Jones & Wang, 2010; Wang, 2016). The COLONY parentage assignment software was utilized as it can produce assignments when the genotype of one of the parents is missing, either due to a missing parental sample, or due to failure to produce a parental genotype from an existing sample. Given that PBT assignments for many potential populations were evaluated for each fishery and escapement sample, COLONY was run with all broodstock sampled during each year as input as a single unit for analysis of fishery and escapement samples, with no differentiation among populations. Although the COLONY assumption of a single population in the parent pool was violated, analysis of known‐origin samples indicated that very high levels of accuracy (100%) were achieved in population assignments when pooling of potential parents in contributing populations was conducted (Beacham et al., 2019a). Two‐parent assignments were accepted only when both assigned parents originated from the same population. Two‐parent and single‐parent assignments were accepted only when the probability of correct assignment was ≥ 0.85 for the parent pair. An additional constraint on the single‐parent assignment before it was accepted was that both the PBT assignment and GSI assignment corresponded to populations in the same CU. Polygamous mating was assumed for the COLONY analysis. Simple pairwise comparisons between offspring and potential parents were conducted. The PBT baseline for individuals sampled in the 2016 hatchery broodstock and nonbroodstock escapements (jacks) and 2017 fisheries and escapements and hatchery broodstocks included all broodstocks sampled in 2014 and 2015, as coho salmon in southern BC are predominately three years of age (Sandercock, 1991). Similarly, the PBT baseline for individuals sampled in 2018 fisheries and hatchery broodstocks included all broodstocks sampled in 2014, 2015, and 2016. Jacks selected in the 2017 escapement were identified via body size, as there is a fairly clear distinction between jack and adult body size within populations (Sandercock, 1991), and subsequently assigned to parents in the 2015 hatchery broodstocks. Individuals with more than 120 missing genotypes were eliminated from further analyses. An estimated genotyping error rate of 1% was used for COLONY assignments. Previously, Beacham et al. (2017) had reported that an average genotyping error rate of 1.07% (1,220 discrepancies in 114,105 comparisons) or an allele error rate of 0.53% (1,220 discrepancies in 228,210 comparisons) was observed over the 304 SNPs scored. The parent pair output file was the basic file used in subsequent analyses.

### 2.5 Geographical concordance

The geographical distribution of the 26 populations for which PBT identifications were available allowed an evaluation of whether a single population in a region displays characteristics that are representative of the other populations within the same local geographic area. Populations were grouped into the following regions; (a) west coast of Vancouver Island (WCVI) (Conuma River, Robertson Creek, Nitinat River); (b) east coast of Vancouver Island (ECVI) (Quinsam River, Trent River, Puntledge River, Big Qualicum River, Rosewall Creek, French Creek, Goldstream River); (c) southern mainland (Tenderfoot Creek, Mamquam River, Capilano River, Seymour River); (d) Lower Fraser River (Inch Creek, Norrish Creek, Stave River, Chehalis River, Chilliwack River, Kanaka Creek); (e) Boundary Bay (Nicomekl River, Serpentine River); (f) Skeena River (Zymacord River); and (g) Thompson River (Salmon River, Eagle River, and Coldwater River). Although the Quinsam River and Qualicum River populations are located in the same CU, it has been traditional to consider Quinsam River as an indicator population for northeastern Vancouver Island populations bordering Johnstone Strait, whereas Qualicum River was considered as an indicator for lower ECVI populations bordering the Strait of Georgia. Subsequent analyses both included and excluded Quinsam River as representative of the ECVI region. The Salmon River, Eagle River, and Coldwater River populations are all located in the Thompson River drainage, but the Salmon River and Eagle River area tributaries of the South Thompson River, and the Coldwater River is a tributary of the lower Thompson River.

Four traits were compared among populations within regions. The first trait was distribution of identifications across the six marine fishing regions noted earlier. Statistical analysis was conducted with Fisher's exact test, and populations were required to display ≥ 5 fishery PBT identifications to be included in the analysis. The second trait investigated was the monthly distribution of marine and freshwater fishery PBT identifications, with months ranging from June through October. Any May identifications were pooled with June, and any November or later identifications were pooled with October. The third trait investigated was the distribution of total progeny sampled by male, with classes set at 0, 1, 2, 3, 4, 5, 6, 7, and ≥ 8 progeny, with the progeny number determined as the sum of PBT identifications in fishery, hatchery broodstock, and escapement samples for each male. As noted by Beacham et al. (2019b), the typical hatchery spawning design was to cross a single male with a single female, but in practice some males produced offspring from more than one female, presumably as a result of carryover of viable sperm in a fertilization bucket even with intervening rinsing and drying between fertilizations. Population distributions were compared statistically via Fisher's exact test. The fourth trait investigated was the distribution of progeny exploitation rate by male, with classes set a 0%, >0‐<20%, 20–<40%, 40–<60%. 60–<80%, and 80%–100%. Exploitation rate was defined as the number of fishery PBT identifications/(fishery PBT identifications + escapement PBT identifications)*100. The analysis was restricted to those males contributing at least four progeny to fisheries and escapement sampling. Population distributions were compared statistically via Fisher's exact test. Populations were excluded from the exploitation rate analysis if no broodstock male displayed at least four subsequent PBT identifications.

## 3 RESULTS

### 3.1 Fishery captures

**FIGURE 2:**
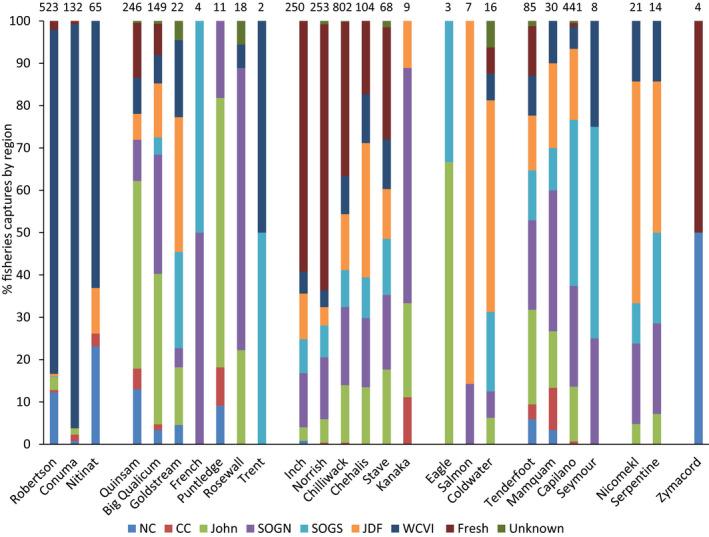
Percentage of 2017 and 2018 PBT fishery identifications by fishery region for 26 populations of coho salmon in British Columbia where the 2014, 2015, and 2016 hatchery broodstocks were genotyped. Populations have been grouped into six geographic regions in British Columbia: west coast of Vancouver Island (Robertson, Nitinat, Conuma); east coast of Vancouver Island (Quinsam, Big Qualicum, Puntledge, Rosewall, Goldstream, French, Trent); Lower Fraser River (Inch, Norrish, Chilliwack, Chehalis, Stave, Kanaka), Thompson River (Coldwater, Salmon, Eagle); Boundary Bay (Nicomekl, Serpentine); and Skeena River (Zymacord). Values above bars are number of fishery PBT identifications per population

The key assumption underlying an assessment method employing indicator populations is that the indicator population (typically coded‐wire‐tagged) displays characteristics that are representative of the other untagged populations within the management unit or geographic region that it is intended to represent. A critical factor is whether or not the indicator population displays a distribution of captures among fishery areas that is similar to other populations in the management unit. There were limited annual differences in fishery identifications within populations, with 2017 and 2018 fishery identifications outlined in Tables 2 and 3. For example, the Robertson Creek population displayed a proportionately more northward migration pattern than other populations, with fishery identifications in NC fisheries, and this pattern was observed both in 2017 (11.6% of identifications) and in 2018 (13.0% of identifications). Conversely, fishery identifications for the Conuma River population, the most northern population on the west coast of Vancouver Island, were virtually all in the WCVI fishery in both 2017 and 2018 (126 of 132 total recoveries, 95.5%). There was little evidence to indicate that populations within a geographic region displayed similar distributions of fishery captures. For example, in the west Vancouver Island CU, there were significant differences in the distribution of fishery PBT identifications among the Robertson Creek and Conuma River populations ( $\chi_{5}^{2}$ = 19.5, *p* < .005), with Robertson Creek displaying proportionately more northern fishery identifications than Conuma River as noted previously (Figure 2). Robertson Creek is the only coded‐wire‐tagged population on the west coast of Vancouver Island and is applied by the Pacific Salmon Commission as an indicator population for the entire west coast of the island. However, there were significant differences in fishery identifications between Robertson Creek and Nitinat River populations ( $\chi_{5}^{2}$ = 31.5, *p* < .001), with more Nitinat River fishery recoveries in the Juan de Fuca Strait fishery (Figure 2). There was little concordance among locations of fishery identifications for the three west coast of Vancouver Island populations ( $\chi_{10}^{2}$ = 79.2, *p* < .001).

**TABLE 2:** PBT percentage assignments by population for fisheries in BC during 2017 for samples sent to a central laboratory for potential CWT recovery

CU	CU ID	Population	Total	NC troll	NC sport	Central troll	Central sport	John	SOGN	SOGS	JDF	WCVI troll	WCVI sport	Alberni	Fresh	Unk
WCVI	CO‐17	Robertson	276	6.2	5.4	0.7	0.0	5.8	0.0	0.4	0.4	0.0	14.1	64.1	2.9	0.0
WCVI	CO‐17	Conuma	14	0.0	0.0	0.0	0.0	14.3	0.0	0.0	0.0	0.0	71.4	14.3	0.0	0.0
JDF	CO‐16	Nitinat	24	0.0	16.7	0.0	0.0	0.0	0.0	0.0	20.8	0.0	54.2	8.3	0.0	0.0
ECVI	CO‐13	Quinsam	119	3.4	0.8	0.0	5.0	58.0	10.1	0.0	7.6	0.0	3.4	0.8	10.9	0.0
ECVI	CO‐13	Qualicum	89	1.1	3.4	2.2	0.0	32.6	32.6	4.5	15.7	0.0	5.6	0.0	2.2	0.0
ECVI	CO‐13	Goldstream	12	0.0	8.3	0.0	0.0	25.0	0.0	16.7	25.0	0.0	25.0	0.0	0.0	0.0
ECVI	CO‐13	Puntledge	4	0.0	0.0	25.0	0.0	50.0	25.0	0.0	0.0	0.0	0.0	0.0	0.0	0.0
ECVI	CO‐13	Rosewall	7	0.0	0.0	0.0	0.0	14.3	85.7	0.0	0.0	0.0	0.0	0.0	0.0	0.0
LF	CO‐47	Inch	121	0.8	0.0	0.0	0.0	4.1	8.3	5.8	14.9	0.0	2.5	0.0	63.6	0.0
LF	CO‐47	Norrish	109	0.0	0.0	0.0	0.0	7.3	17.4	10.1	8.3	0.0	2.8	0.0	54.1	0.0
LF	CO‐47	Chilliwack	319	0.0	0.0	0.0	0.3	8.2	22.3	5.6	21.0	0.0	4.7	0.0	37.9	0.0
LF	CO‐47	Chehalis	70	0.0	0.0	0.0	0.0	12.9	11.4	8.6	38.6	0.0	8.6	0.0	20.0	0.0
LF	CO‐47	Stave	32	0.0	0.0	0.0	0.0	15.6	6.3	12.5	18.8	0.0	0.0	3.1	43.8	0.0
ST	CO‐8	Salmon	6	0.0	0.0	0.0	0.0	0.0	16.7	0.0	83.3	0.0	0.0	0.0	0.0	0.0
LT	CO‐7	Coldwater	10	0.0	0.0	0.0	0.0	10.0	10.0	20.0	50.0	0.0	10.0	0.0	0.0	0.0
HSBI	CO‐10	Tenderfoot	49	0.0	0.0	0.0	6.1	24.5	14.3	12.2	18.4	0.0	2.0	0.0	20.4	2.0
HSBI	CO‐10	Mamquam	17	0.0	0.0	5.9	11.8	23.5	23.5	11.8	23.5	0.0	0.0	0.0	0.0	0.0
HSBI	CO‐10	Capilano	202	0.0	0.0	0.0	1.0	17.8	25.2	36.6	12.4	0.0	5.0	0.5	1.5	0.0
BB	CO‐1	Nicomekl	7	0.0	0.0	0.0	0.0	0.0	0.0	0.0	100.0	0.0	0.0	0.0	0.0	0.0
BB	CO‐1	Serpentine	12	0.0	0.0	0.0	0.0	8.3	16.7	16.7	41.7	0.0	16.7	0.0	0.0	0.0

Note

Conservation units (CUs) and CU number (CU ID) were west coast of Vancouver Island (WCVI), Juan de Fuca‐Pachena (JDF), east coast of Vancouver Island–Georgia Strait (ECVI), Lower Fraser River (LF), South Thompson River (ST), Lower Thompson River (LT), Howe Sound–Burrard Inlet (HSBI), Boundary Bay (BB), and Lower Skeena River (LS). Fisheries were northern troll (NC), northern sport, central troll, central sport, Johnstone Strait sport (John), Strait of Georgia north sport (SOGN), Strait of Georgia south sport (SOGS), Juan de Fuca Strait sport (JDF), west coast of Vancouver Island sport and troll (WCVI), Barkley Sound and Alberni Inlet sport, freshwater sport (Fresh), and unknown location (Unk). Total is the total number of fishery PBT identifications for the population.

**TABLE 3:** PBT percentage assignments by population for fisheries in BC during 2018 for samples sent to a central laboratory for potential CWT recovery

CU	CU ID	Population	Total	NC troll	NC sport	Central troll	Central sport	John	SOGN	SOGS	JDF	WCVI troll	WCVI sport	Alberni	Fresh	Unk
WCVI	CO‐17	Robertson	247	7.7	5.3	0.0	0.4	0.0	0.0	0.0	0.8	4.5	41.7	38.5	1.2	0.0
WCVI	CO‐17	Conuma	118	0.0	0.8	0.0	1.7	0.0	0.0	0.0	0.0	5.1	91.5	0.0	0.8	0.0
JDF	CO‐16	Nitinat	41	0.0	26.8	0.0	4.9	0.0	0.0	0.0	4.9	17.1	46.3	0.0	0.0	0.0
ECVI	CO‐13	Quinsam	127	13.4	7.9	0.0	4.7	31.5	9.4	0.0	4.7	2.4	10.2	0.0	15.0	0.8
ECVI	CO‐13	Qualicum	60	1.7	0.0	0.0	0.0	40.0	21.7	3.3	8.3	1.7	6.7	0.0	15.0	1.7
ECVI	CO‐13	Goldstream	10	0.0	0.0	0.0	0.0	0.0	10.0	30.0	40.0	10.0	0.0	0.0	0.0	10.0
ECVI	CO‐13	French	4	0.0	0.0	0.0	0.0	0.0	50.0	50.0	0.0	0.0	0.0	0.0	0.0	0.0
ECVI	CO‐13	Puntledge	7	14.3	0.0	0.0	0.0	71.4	14.3	0.0	0.0	0.0	0.0	0.0	0.0	0.0
ECVI	CO‐13	Rosewall	11	0.0	0.0	0.0	0.0	27.3	54.5	0.0	0.0	0.0	9.1	0.0	0.0	9.1
ECVI	CO‐13	Trent	2	0.0	0.0		0.0	0.0	0.0	50.0	0.0	0.0	50.0	0.0	0.0	0.0
LF	CO‐47	Inch	129	0.0	0.8	0.0	0.0	2.3	17.1	10.1	7.0	0.0	7.8	0.0	55.0	0.0
LF	CO‐47	Norrish	144	0.0	0.0	0.0	0.7	4.2	12.5	5.6	1.4	0.0	4.9	0.0	69.4	1.4
LF	CO‐47	Chilliwack	483	0.0	0.0	0.0	0.4	17.2	15.9	10.8	8.1	0.8	11.0	0.0	35.4	0.4
LF	CO‐47	Chehalis	34	0.0	0.0	0.0	0.0	14.7	26.5	11.8	17.6	0.0	17.6	0.0	11.8	0.0
LF	CO‐47	Stave	36	0.0	0.0	0.0	0.0	19.4	27.8	13.9	5.6	2.8	16.7	0.0	11.1	2.8
LF	CO‐47	Coquitlam	1	0.0	0.0	0.0	0.0	0.0	0.0	0.0	0.0	0.0	100.0	0.0	0.0	0.0
LF	CO‐47	Kanaka	9	0.0	0.0	0.0	11.1	22.2	55.6	0.0	11.1	0.0	0.0	0.0	0.0	0.0
ST	CO‐8	Eagle	3	0.0	0.0	0.0	0.0	66.7	0.0	33.3	0.0	0.0	0.0	0.0	0.0	0.0
ST	CO‐8	Salmon	1	0.0	0.0	0.0	0.0	0.0	0.0	0.0	100.0	0.0	0.0	0.0	0.0	0.0
LT	CO‐7	Coldwater	6	0.0	0.0	0.0	0.0	0.0	0.0	16.7	50.0	0.0	0.0	0.0	16.7	16.7
HSBI	CO‐10	Tenderfoot	36	0.0	13.9	0.0	0.0	19.4	30.6	11.1	5.6	5.6	13.9	0.0	0.0	0.0
HSBI	CO‐10	Mamquam	13	0.0	7.7	0.0	0.0	0.0	46.2	7.7	15.4	0.0	15.4	7.7	0.0	0.0
HSBI	CO‐10	Capilano	239	0.0	0.0	0.0	0.4	8.8	22.6	41.4	20.5	0.4	4.6	0.0	0.4	0.8
HSBI	CO‐10	Seymour	8	0.0	0.0	0.0	0.0	0.0	25.0	50.0	0.0	0.0	25.0	0.0	0.0	0.0
BB	CO‐1	Nicomekl	14	0.0	0.0	0.0	0.0	7.1	28.6	14.3	28.6	0.0	21.4	0.0	0.0	0.0
BB	CO‐1	Serpentine	2	0.0	0.0	0.0	0.0	0.0	50.0	50.0	0.0	0.0	0.0	0.0	0.0	0.0
LS	CO‐32	Zymacord	4	50.0	0.0	0.0	0.0	0.0	0.0	0.0	0.0	0.0	0.0	0.0	50.0	0.0

Note

Conservation units (CUs) were west coast of Vancouver Island (WCVI), Juan de Fuca‐Pachena (JDF), east coast of Vancouver Island–Georgia Strait (ECVI), Lower Fraser River (LF), South Thompson River (ST), Lower Thompson River (LT), Howe Sound–Burrard Inlet (HSBI), Boundary Bay (BB), and Lower Skeena River (LS). Fisheries were northern troll, northern sport, central troll, central sport, Johnstone Strait sport (John), Strait of Georgia north sport (SOGN), Strait of Georgia south sport (SOGS), Juan de Fuca Strait sport (JDF), west coast of Vancouver Island sport and troll (WCVI), Barkley Sound and Alberni Inlet sport, freshwater sport (Fresh), and unknown location (Unk). Total is the total number of fishery PBT identifications for the population.

In the east Vancouver Island–Georgia Strait CU, there were significant differences among the five populations retained in the analysis (Quinsam, Big Qualicum, Puntledge, Rosewall, Goldstream) $\chi_{24}^{2}$ = 133.0, *p* < .001), and between Quinsam River and Big Qualicum River ( $\chi_{6}^{2}$ = 47.2, *p* < .001), the two geographically most proximal populations. The Quinsam River population contributed progeny disproportionately to the NC and JS fisheries compared with the other populations, and the Goldstream River population contributed disproportionately to the JDF and WCVI fisheries (Figure 2). With Quinsam River excluded from the analysis, there were still significant differences in the distribution of fishery identifications for ECVI populations ( $\chi_{18}^{2}$ = 53.4, *p* < .001). Quinsam River, Qualicum River, and Puntledge River populations are all coded‐wire‐tagged.

In the Lower Fraser River CU, there were significant differences in marine fishery identifications among the six populations in the analysis ( $\chi_{30}^{2}$ = 77.9, *p* < .001). Fishery identifications of the Chehalis River population were proportionately more prevalent in the JDF fishery, whereas those of the Stave River population were more prevalent in the JS and northern GS fisheries (Figure 2). The Inch Creek population is the only coded‐wire‐tagged population in the CU, and its distribution of fishery identifications was significantly different from those of the Chilliwack River ( $\chi_{6}^{2}$ = 22.2, *p* < .001) and Kanaka Creek populations ( $\chi_{6}^{2}$ = 18.3, *p* < .01), and approached significance for the Chehalis River ( $\chi_{5}^{2}$ = 10.9, *p* < .06), Stave River ( $\chi_{5}^{2}$ = 10.2, *p* < .06), and Norrish Creek ( $\chi_{6}^{2}$ = 11.7, *p* < .07) populations.

In the Howe Sound–Burrard Inlet CU, located on the southern BC mainland, there were differences in fishery recoveries of progeny from the four populations in the CU ( $\chi_{18}^{2}$ = 82.8, *p* < .001), with only the Seymour River population coded‐wire tagged in CU. The Tenderfoot Creek and Mamquam River populations contributed proportionately more to the CC and JS fisheries, whereas the Capilano River and Seymour River populations contributed proportionately more to the GS fishery (Figure 2).

In the Boundary Bay CU, there was no significant difference in the distribution of fishery identifications between the two populations in the CU (*p* > .10) (Figure 2). In summary, there was little evidence to support the assumption that a single population chosen as an indicator in a CU is likely to display a distribution of captures among fishery areas that is representative of other populations in the CU.

### 3.2 Timing of captures

**FIGURE 3:**
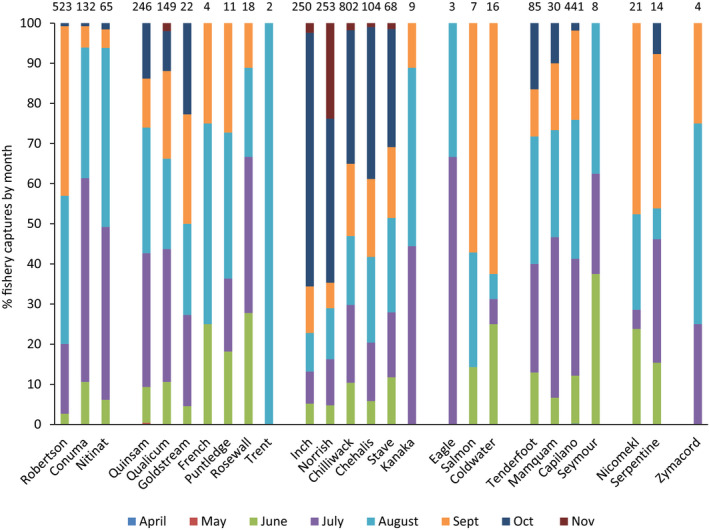
Percentage of 2017 and 2018 PBT fishery identifications by month for 26 populations of coho salmon in British Columbia. Populations were grouped into the geographic regions as outlined in Figure 2. Values above bars are number of fishery PBT identifications per population

The month of fishery capture was also evaluated for conformance among populations within a CU, with the timing of 2017 and 2018 fishery identifications outlined in Tables 4 and 5. In the west Vancouver Island CU, there were significant differences in the monthly distribution of fishery PBT identifications among the Robertson Creek and Conuma River populations ( $\chi_{4}^{2}$ = 101.0, *p* < .001), with the Robertson Creek population displaying proportionately more September fishery identifications (42.2%) than Conuma River (5.3%) (Figure 3). For the WCVI region as a whole, the monthly timing of fishery identifications was similar between the Nitinat River and Conuma River populations, but quite distinct from that of the Robertson Creek population (Figure 3).

**TABLE 4:** PBT percentage assignments by population and month for fisheries in BC during 2017 for samples sent to a central laboratory for potential CWT recovery

CU	CU ID	Population	Total	April	May	June	July	August	September	October
WCVI	CO‐17	Robertson	276	0.0	0.0	2.9	12.3	40.6	42.8	1.4
WCVI	CO‐17	Conuma	14	0.0	0.0	7.1	7.1	71.4	7.1	7.1
JDF	CO‐16	Nitinat	24	0.0	0.0	4.2	20.8	58.3	12.5	4.2
ECVI	CO‐13	Quinsam	119	0.0	0.0	7.6	35.3	29.4	16.8	10.9
ECVI	CO‐13	Qualicum	89	0.0	0.0	7.9	33.7	27.0	21.3	10.1
ECVI	CO‐13	Goldstream	12	0.0	0.0	8.3	33.3	25.0	16.7	16.7
ECVI	CO‐13	Puntledge	4	0.0	0.0	0.0	25.0	75.0	0.0	0.0
ECVI	CO‐13	Rosewall	7	0.0	0.0	28.6	42.9	14.3	14.3	0.0
LF	CO‐47	Inch	121	0.0	0.0	2.5	4.1	5.0	13.2	75.2
LF	CO‐47	Norrish	109	0.0	0.0	4.6	15.6	10.1	11.0	58.7
LF	CO‐47	Chilliwack	319	0.0	0.0	3.1	18.5	13.5	19.7	45.1
LF	CO‐47	Chehalis	70	0.0	0.0	0.0	17.1	17.1	15.7	50.0
LF	CO‐47	Stave	32	0.0	0.0	6.3	3.1	15.6	21.9	53.1
ST	CO‐8	Salmon	6	0.0	0.0	16.7	0.0	33.3	50.0	0.0
LT	CO‐7	Coldwater	10	0.0	0.0	20.0	10.0	10.0	60.0	0.0
HSBI	CO‐10	Tenderfoot	49	0.0	0.0	8.2	30.6	20.4	12.2	28.6
HSBI	CO‐10	Mamquam	17	0.0	0.0	5.9	35.3	29.4	11.8	17.6
HSBI	CO‐10	Capilano	202	0.0	0.0	9.1	26.4	33.5	28.4	5.1
BB	CO‐1	Nicomekl	7	0.0	0.0	14.3	0.0	0.0	85.7	0.0
BB	CO‐1	Serpentine	12	0.0	0.0	16.7	16.7	8.3	50.0	8.3

Note

Conservation units (CUs) were west coast of Vancouver Island (WCVI), Juan de Fuca–Pachena (JDF), east coast of Vancouver Island–Georgia Strait (ECVI), Lower Fraser River (LF), South Thompson River (ST), Lower Thompson River (LT), Howe Sound–Burrard Inlet (HSBI), and Boundary Bay (BB). Total is the total number of fishery PBT identifications for the population.

**TABLE 5:** PBT percentage assignments by population and month for fisheries in BC during 2017 for samples sent to a central laboratory for potential CWT recovery

CU	CU ID	Population	Total	April	May	June	July	August	September	October	November
WCVI	CO‐17	Robertson	247	0.0	0.0	2.4	23.1	32.8	41.7	0.0	0.0
WCVI	CO‐17	Conuma	118	0.0	0.0	11.0	55.9	28.0	5.1	0.0	0.0
JDF	CO‐16	Nitinat	41	0.0	0.0	7.3	56.1	36.6	0.0	0.0	0.0
ECVI	CO‐13	Quinsam	127	0.0	0.8	10.2	31.5	33.1	7.9	16.5	0.0
ECVI	CO‐13	Qualicum	60	0.0	0.0	15.0	31.7	15.0	23.3	10.0	5.0
ECVI	CO‐13	Goldstream	10	0.0	0.0	0.0	10.0	20.0	40.0	30.0	0.0
ECVI	CO‐13	French	4	0.0	0.0	25.0	0.0	50.0	25.0	0.0	0.0
ECVI	CO‐13	Puntledge	7	0.0	0.0	28.6	14.3	14.3	42.9	0.0	0.0
ECVI	CO‐13	Rosewall	11	0.0	0.0	27.3	36.4	27.3	9.1	0.0	0.0
ECVI	CO‐13	Trent	2	0.0	0.0	0.0	0.0	100.0	0.0	0.0	0.0
LF	CO‐47	Inch	129	0.0	0.0	7.8	11.6	14.0	10.1	51.9	4.7
LF	CO−47	Norrish	144	0.0	0.0	4.9	8.3	14.6	2.8	27.8	41.7
LF	CO‐47	Chilliwack	483	0.0	0.2	14.9	19.9	19.7	17.0	25.5	2.9
LF	CO‐47	Chehalis	34	0.0	0.0	17.6	8.8	29.4	26.5	14.7	2.9
LF	CO‐47	Stave	36	0.0	0.0	16.7	27.8	30.6	13.9	8.3	2.8
LF	CO‐47	Coquitlam	1	0.0	0.0	0.0	100.0	0.0	0.0	0.0	0.0
LF	CO‐47	Kanaka	9	0.0	0.0	0.0	44.4	44.4	11.1	0.0	0.0
ST	CO‐8	Eagle	3	0.0	0.0	0.0	66.7	33.3	0.0	0.0	0.0
ST	CO‐8	Salmon	1	0.0	0.0	0.0	0.0	0.0	100.0	0.0	0.0
LT	CO‐7	Coldwater	6	0.0	0.0	33.3	0.0	0.0	66.7	0.0	0.0
HSBI	CO‐10	Tenderfoot	36	0.0	0.0	19.4	22.2	47.2	11.1	0.0	0.0
HSBI	CO‐10	Mamquam	13	0.0	0.0	7.7	46.2	23.1	23.1	0.0	0.0
HSBI	CO‐10	Capilano	239	0.4	0.0	14.2	31.4	35.6	17.2	1.3	0.0
HSBI	CO‐10	Seymour	8	0.0	0.0	37.5	25.0	37.5	0.0	0.0	0.0
BB	CO‐1	Nicomekl	14	0.0	0.0	28.6	7.1	35.7	28.6	0.0	0.0
BB	CO‐1	Serpentine	2	0.0	0.0	0.0	100.0	0.0	0.0	0.0	0.0
LS	CO‐32	Zymacord	4	0.0	0.0	0.0	25.0	50.0	25.0	0.0	0.0

Note

Conservation units (CUs) were west coast of Vancouver Island (WCVI), Juan de Fuca–Pachena (JDF), east coast of Vancouver Island–Georgia Strait (ECVI), Lower Fraser River (LF), South Thompson River (ST), Lower Thompson River (LT), Howe Sound–Burrard Inlet (HSBI), Boundary Bay (BB), and Lower Skeena River (LS). Total is the total number of fishery PBT identifications for the population.

In the east Vancouver Island–Georgia Strait CU, there were no significant differences in timing of monthly fishery identifications among the five populations retained in the analysis (Figure 3). July and August were the main months of fishery identifications.

In the Lower Fraser River CU, there was substantial differentiation in the monthly distribution of fishery progeny captures among populations ( $\chi_{25}^{2}$ = 308.0, *p* < .001). Over 60% of the Inch Creek fishery progeny captures were observed in the October freshwater fishery, whereas those for the other populations ranged between 11% and 41% (Figure 3). November identifications for the Norrish Creek population accounted for 24% of all identifications and occurred entirely in the freshwater fishery, reflective of the later timing of return of this population in comparison with the Inch Creek population.

In the Howe Sound–Burrard Inlet CU, there were significant differences in the monthly distribution of fishery identifications among the four populations in the region ( $\chi_{12}^{2}$ = 38.3, *p* < .001), with higher percentages of Capilano River identifications occurring in August and September fisheries (57%) than in the other populations (37%–43%) (Figure 3).

In the Boundary Bay CU, there was no significant differentiation between populations in the CU with respect to monthly fishery progeny captures (*p* > .10). September was the dominant month of fishery captures for both populations (Figure 3).

### 3.3 Productivity

**FIGURE 4:**
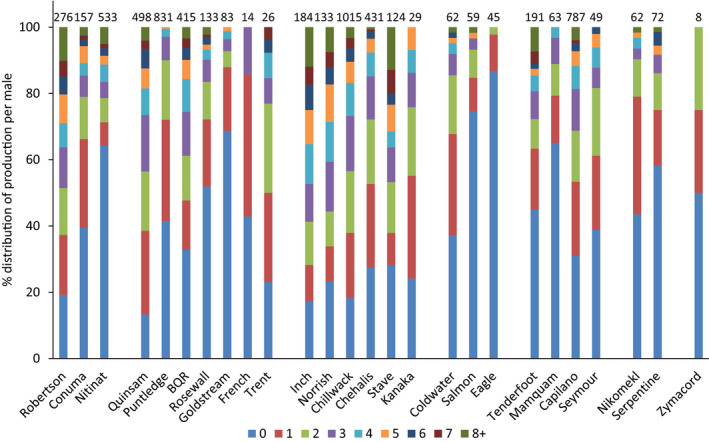
Percentage distribution of total PBT identifications by male for 2017–2018 fishery and 2016–2018 hatchery broodstock and escapements. PBT identifications ≥ 8 for males in a population were grouped into a single class. Values above bars are number of males genotyped per population

The number of progeny contributed per male was investigated among populations within the geographic regions outlined, as the actual distribution of male productivity is a key metric to compare among populations with an assumed similar distribution. In the WCVI region, there were significant differences in the distribution of progeny contributions per male among the three populations in the region ( $\chi_{16}^{2}$ = 189.0, *p* < .001), with the Nitinat River population less productive than other populations in the region (Figure 4). In the ECVI unit, there were significant differences among the seven populations in the analysis ( $\chi_{48}^{2}$ = 402.4, *p* < .001), with the Puntledge River and Rosewall Creek populations less productive than the Quinsam River and Big Qualicum River populations (Figure 4). With the Quinsam River population removed from the analysis, there were significant differences among the remaining six populations in the analysis ( $\chi_{40}^{2}$ = 259.3, *p* < .001). In the southern mainland region, there were significant differences in the distribution of male total progeny among the four populations in the region ( $\chi_{24}^{2}$ = 68.1, *p* < .001), with the Capilano River population more productive than the other three populations in the region (Figure 4). In the Lower Fraser River region, there were clear differences in the distribution of total progeny distributions by male among the six populations ( $\chi_{40}^{2}$ = 176.8, *p* < .001), with Inch Creek and Stave River among the more productive populations (Figure 4). In Boundary Bay, there were restricted number of progeny observed for the two populations, and there was no significant difference in the distribution of total progeny per male between the two populations ( $\chi_{7}^{2}$ = 11.7, *p* > .10) (Figure 4). Similarly, with a restricted total number of progeny identified per male for the two south Thompson River populations (Salmon River, Eagle River), there was no significant difference in the distribution of total progeny per male between the two populations ( $\chi_{5}^{2}$ = 5.3, *p* > .10) (Figure 4). However, when the lower Thompson River population (Coldwater River) was included, there was a significant difference among the three Thompson River drainage populations ( $\chi_{14}^{2}$ = 36.3, *p* < .001). Overall, there was little evidence to indicate that populations within a geographic region displayed similar distributions of male productivity.

### 3.4 Exploitation rate

**FIGURE 5:**
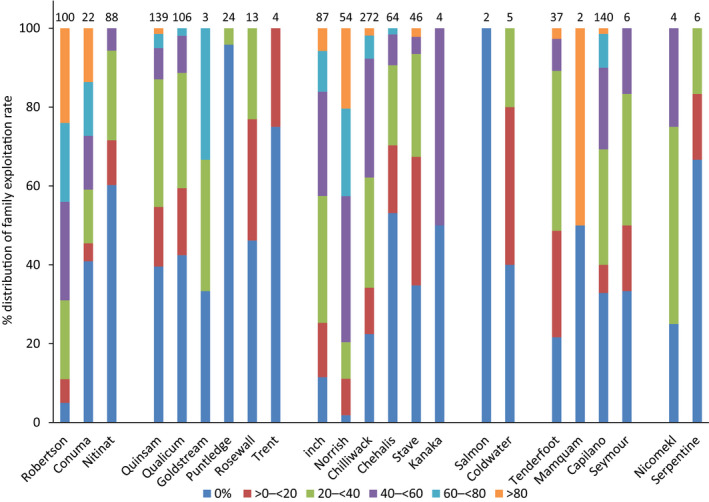
Percentage distribution of family exploitation rates for populations of coho salmon, with exploitation rate defined as (the number of PBT fishery identifications for an individual male/the sum of fishery and escapement PBT identifications for the male)*100. The analysis was restricted to those families with at least four total PBT identifications. Values above bars are number of males with at least four PBT identifications per population

Estimation of exploitation rate is a key function of an indicator population, as extrapolation of this estimated exploitation rate to other unmarked populations in the management unit or geographic region is a key tenet of status assessment via CWTs. In the WCVI region, there was substantial differentiation in the distribution of exploitation rate across males among populations within the region ( $\chi_{10}^{2}$ = 97.6, *p* < .001), with the exploitation profile of the Robertson Creek population unlike that of other populations in the region (Figure 5). In the ECVI CU, there was a clear differentiation between the exploitation rate across males for the Quinsam River and Big Qualicum River populations compared with other populations in the CU ( $\chi_{25}^{2}$ = 47.1, *p* < .01) (Figure 5). With the Quinsam River population removed from the analysis, differentiation in exploitation rate among ECVI populations was still observed ( $\chi_{16}^{2}$ = 43.9, *p* < .001). In the southern mainland region, substantial differentiation in exploitation rate across males was observed among populations ( $\chi_{15}^{2}$ = 42.4, *p* < .001) (Figure 5). In the Lower Fraser River region, there was substantial differentiation in the distribution of exploitation rates across males among populations ( $\chi_{25}^{2}$ = 155.8, *p* < .001). The exploitation profile of the Inch Creek population could not be reliably extrapolated to represent other populations in the region (Figure 5). Limited sample size precluded analyses of exploitation rate across males for other regions. Overall, there was little evidence to indicate that exploitation rates on populations within a geographic region were homogenous.

## 4 DISCUSSION

The currently accepted method for assessment and management for coho salmon fisheries and hatchery and naturally spawning populations in BC is based upon CWTs, a technique initially applied in the 1970s and later constituted the main assessment method under the Pacific Salmon Treaty signed between Canada and the United States of America in 1985. There are currently 44 CUs designated for coho salmon in BC under Canada's Wild Salmon Policy (WSP), and given the limited distribution of coho salmon populations marked with CWTs in BC, CWTs cannot provide the CU resolution for wild population assessment, nor a management unit resolution for hatchery and fishery assessment and management. The successful application of PBT and GSI as outlined by Beacham et al. (2019a) allowed accurate identification of coho salmon sampled from mixed‐stock fisheries and enabled the first Canadian assessment of fishery impacts that was sufficiently informative for conservation‐based management as envisaged in the WSP. In the present study, we provided information on indicator populations that was derived solely from the application of PBT, and can be considered as delivering additional or novel information, not provided by the existing CWT system, to inform management of fisheries and hatchery broodstocks for coho salmon.

### 4.1 Indicator populations

Coho salmon CWT indicator populations, established at a time when the fine‐scale biodiversity within and among proximal salmon populations was poorly characterized, form the basis of assessment in the current CWT program. However, the CWT regions are in many cases not well‐aligned with CU boundaries, with many CUs lacking CWT indicator populations entirely and others containing multiple tagged populations. Moreover, the current study has demonstrated that the assumed biological homogeneity within and among populations of an indicator region may often not exist, as heterogeneity in fishery recoveries among proximal hatchery populations of coho salmon within a CU was observed. This undermines the key assumption that the marine migration pattern and associated ocean distribution of populations, and therefore fishery recoveries, are similar across populations within a CU. However, the data derived from the study were fishery‐dependent, subject to any biases occurring due to location and timing of fishery sampling and possibly unrepresentative sampling. These results are in contrast to those reported previously by Weitkamp and Neely (2002) for coho salmon, who suggested that recovery patterns for tagged wild populations were consistent with those of hatchery populations from the same region and that marine distributions based on hatchery populations are reasonable proxies for marine distributions of wild populations from the same local area. Part of the difference between the studies may relate to the smaller fishery recovery areas in southern BC employed in the current study in comparison with those of Weitkamp and Neely (2002), as well as a more extensive regional coverage for southern BC populations in the current study.

Estimation of age‐specific exploitation rate is one of the more important outputs of the CWT assessment method, but we found significant differences in family‐specific exploitation rates among populations within small geographic regions, putting into question the assumption that the exploitation rate of indicator populations will be representative of other populations in the management unit. Age‐specific exploitation rates are of more management concern in Chinook salmon than in coho salmon. Satterthwaite et al. (2014) and Satterthwaite and O’Farrell (2018) compared fishery distributions of a tagged indicator population with those of other populations via genetic stock identification and reported that representativeness of the indicator population for estimation of fishery interceptions of other populations was dependent upon the timing of the fishery. Thus, an indicator population may be representative of other populations in a region under a very specific set of circumstances.

Variability in exploitation rates in Canadian fisheries arose from the increasing importance of terminal fisheries (fisheries that occur near the river mouth) that result in differing exploitation rates on geographically proximate populations. For example, the Robertson Creek population is considered as an indicator population, but it is subjected to terminal exploitation in the Barkley Sound and Alberni Canal region, unlike the Conuma River and Nitinat River populations. Exploitation rates for the Robertson Creek population can be calculated with the terminal areas excluded, but that would make the Robertson Creek population unrepresentative of other coho salmon populations within Barkley Sound (which include many wild populations not supported by hatcheries). Moreover, removal of terminal harvest of the Robertson Creek population increased the proportion of samples recovered from northern fisheries (28%) for this population, similar to the Nitinat River population (26%), but substantially larger than the 1% of displayed by the Conuma River population.

Substantial population differences in productivity were observed among populations in a geographic region. As outlined by Beacham et al. (2019b), water temperatures at the Puntledge River hatchery become too high during the summer for juvenile rearing, so approximately 85% of the production from the 2014 and 2015 broodstocks was released as fry and was not adipose‐fin‐clipped. Fry survival would be lower than for smolts released from other EVCI hatcheries, potentially accounting for the lower productivity of the population. Similarly, the apparent poor productivity of the Nitinat River hatchery was accounted for by the culling of approximately 70% of the 1.2 million eggs taken due to high levels of bacterial kidney disease infection rates in female parents, the highest rate observed in any of the populations screened (P. Ackerman, Fisheries and Oceans Canada, pers. comm). Like the Puntledge River population, most of the Nitinat River production was released as fry, leading to lower observed productivity.

In the east coast of Vancouver Island–Georgia Strait CU, the Quinsam River and Qualicum River populations are considered as indicator populations by the Pacific Salmon Commission Coho Technical Committee, but representing ECVI populations adjacent to Johnstone Strait and the Strait of Georgia, respectively. Quinsam River and Qualicum River populations are expected to display differential recoveries from JDF and WCVI fishery locations, as Quinsam River is in a more northerly location than Qualicum River, and coho salmon from Qualicum River are potentially more likely to be caught in more southern fisheries such as the JDF fishery. The populations are within a single CU, but neither of the indicator populations is representative of the Goldstream River population, where 50% of fishery PBT identifications were observed in JDF and WCVI fisheries, compared with 20% for Qualicum River and 13% for Quinsam River populations. The Goldstream population was historically maintained as an important indicator of exploitation rate in US fisheries in Puget Sound.

The evaluation of the representativeness of indicator populations only became possible with the advent of direct DNA sequencing coupled with PBT methodology because the cost of tagging makes the maintenance of multiple tagged populations within CUs prohibitive. Additionally, there exist small wild coho salmon indicator populations, in which emigrating smolts are tagged for fishery and escapement monitoring. PBT methodologies can also be applied to wild populations (Ford, Pearsons, & Murdoch, 2015) and could provide valuable additional information such as the source of hatchery strays in wild populations. Increasingly, the environmental, genetic, and epigenetic impact of hatchery fish on wild populations is becoming of concern with respect to wild population conservation. PBT provides the potential for realizing numerous goals in Canada's WSP (Fisheries and Oceans Canada (FOC) 2018) by enabling assessment on a CU and management unit basis using new assessment methods.

## 5 SUMMARY

Beacham et al. (2019a) demonstrated the potential for implementation of a comprehensive PBT‐GSI methodology for management and assessment of coho salmon in BC, thereby providing a method for assessment of coho salmon by CU, a requirement for implementation of management of wild populations as mandated by Canada's WSP for Pacific salmon. The current study expanded those results by outlining additional or novel information, not provided by the existing CWT system (Pacific Salmon Commission (PSC) 2015), but provided via PBT to inform management of fisheries and hatchery broodstocks for coho salmon. Current coho salmon conservation and enhancement efforts in Canada require comprehensive evaluation and possible modification that cannot be achieved under the current assessment system. Application of PBT analytical methods, powered by direct DNA sequencing, provided evidence that a genetic‐based approach is poised to aid in assessment and management of hatchery origin and wild Pacific salmon in BC. The basic concept of an indicator population, fundamental to the operation of the current assessment programs in BC, was not supported by the observed data derived from PBT analysis for coho salmon populations in BC. Thus, delivery of CU‐based salmon management for biodiversity and sustainability would benefit from consideration of genetic tools now available to characterize, monitor, and conserve biodiversity.

## CONFLICT OF INTEREST

None declared.

## AUTHOR CONTRIBUTION

**Terry D. Beacham:** Conceptualization (lead); Formal analysis (lead); Funding acquisition (lead); Project administration (lead); Supervision (lead); Writing‐original draft (lead); Writing‐review & editing (lead). **Colin Wallace:** Data curation (lead); Formal analysis (equal); Investigation (equal); Methodology (equal); Resources (equal); Software (lead); Validation (equal); Visualization (equal). **Kim Jonsen:** Methodology (equal); Resources (equal); Validation (equal). **Brenda McIntosh:** Methodology (equal); Resources (equal); Validation (equal). **John R. Candy:** Data curation (equal); Methodology (equal); Resources (equal); Visualization (equal). **David Willis:** Methodology (equal); Resources (equal); Visualization (equal). **Cheryl Lynch:** Methodology (equal); Resources (equal); Visualization (equal). **Ruth E. Withler:** Funding acquisition (equal); Writing‐original draft (equal); Writing‐review & editing (equal).

## Data Availability

Multilocus genotypes for all 2016 sampled jacks, as well as individuals in 2017 fishery and escapement samples, are available at DRYAD doi identified as follows: data from: Comparison of coded‐wire tagging with parentage‐based tagging and genetic stock identification in a large‐scale coho salmon fisheries application in British Columbia, Canada. Journal: Evolutionary Applications Provisional https://doi.org/10.5061/dryad.6c31bf2 Data files: 2016_Coho_Jacks_rubias. 2016‐17_Coho_Fishery_Samples_rubias, 2017_Coho_Escapement_rubias. Multilocus genotypes for all sampled jacks, as well as individuals in 2017 fishery and hatchery broodstock samples, are available at DRYAD https://doi.org/10.5061/dryad.6c31bf2 Data files: _Coho_Fishery_Samples_rubias, 2017_Coho_Escapement_rubias. Multilocus genotypes for 2017 hatchery broodstocks are deposited in the Data package title: data from: Variation in migration pattern, broodstock origin, and family productivity of coho salmon hatchery populations in British Columbia, Canada, derived from parentage‐based tagging. Journal: Ecology and Evolution Provisional https://doi.org/10.5061/dryad.3g1r4v3 Data files: 2017 Coho PBT Baseline. Multilocus genotypes for all 2018 sampled jacks, as well as individuals in 2018 fishery and escapement samples, are available at DRYAD doi identified as follows: Insights on the concept of indicator populations derived from parentage‐based tagging in a large‐scale coho salmon application in British Columbia, CanadaProvisional DOI: doi: to be determined Data files: 2018_Coho_Fishery_Samples_rubias, 2018_Coho_Hatchery_broodstock_rubias.
